# Long‐Term Treatment With Juvéderm Hyaluronic Acid Injectables for ≥ 10 Years: Expert Considerations for Natural Outcomes

**DOI:** 10.1111/jocd.71015

**Published:** 2026-06-28

**Authors:** Yuexing Song, Rami Abadi, Gabriela Cella, Stefania Roberts, Fernando Urdiales‐Gálvez, Steve G. Yoelin, Carola de la Guardia

**Affiliations:** ^1^ Department of Plastic and Aesthetic Maxillofacial Surgery The First Affiliated Hospital of Xi'an Jiaotong University Xi'an China; ^2^ RA Clinic Beirut Lebanon; ^3^ Skin Center Rosario Rosario Argentina; ^4^ Private Practice Melbourne Victoria Australia; ^5^ Instituto Medico Miramar Malaga Spain; ^6^ Medical Associates, Inc. Newport Beach California USA; ^7^ Global Aesthetics Medical Affairs Allergan Aesthetics, an AbbVie Company Madrid Spain

**Keywords:** hyaluronic acid, Hylacross, Juvéderm, long‐term treatment, natural results, Vycross

## Abstract

**Background:**

Juvéderm is one of the most widely studied and broadly used hyaluronic acid (HA) product portfolios worldwide. Long‐term treatment can contribute greatly to positive aging, but clinical data over many cycles of injection are currently lacking. Following key principles of best practice can help to ensure natural outcomes are maintained over time.

**Aims:**

To provide clinical expert considerations for long‐term and multiple treatments with Juvéderm, and present case studies treated for ≥ 10 years demonstrating effective and natural outcomes.

**Methods:**

Six clinicians with various specialties and extensive experience with Juvéderm completed a written questionnaire. Their collective clinical expertise and experience form the basis of these considerations.

**Results:**

Maintenance of natural results over long‐term Juvéderm treatment may be facilitated by: (i) strong technical fundamentals (e.g., optimized injection technique and in‐depth anatomical knowledge); (ii) correct product selection (e.g., understanding their composition, properties, preferred injection depths, and key clinical data); and (iii) long‐term planning (e.g., thorough patient assessment, consideration of the whole face, use of an initially conservative approach, combining Juvéderm products with other treatments in an individualized and multimodal strategy, and tracking appearance over time).

**Conclusions:**

Following these key principles of best practice may help clinicians to achieve well‐tolerated and natural outcomes over long‐term treatment with Juvéderm HA injectables.

## 1 Introduction

Injectable hyaluronic acid (HA) products are a mainstay of nonsurgical aesthetic practice [1, 2], and rank among the most highly studied and trusted treatments worldwide. They deliver immediate results and offer a range of other potential advantages that make them popular with patients and practitioners alike—including ease of administration, rapid recovery time, biocompatibility, biodegradability, reversibility with hyaluronidase, and a low risk of major complications compared with surgery [3, 4, 5]. Some of these products can also provide structure with a minimally invasive technique.

The Juvéderm family of products (Allergan Aesthetics, an AbbVie company, Pringy, Annecy, France) is one of the most broadly used and studied portfolios of HA injectables, with more than 40 clinical trials completed globally to date. It comprises two separate technologies: Hylacross [6] and Vycross [7]. Hylacross products are based on high‐molecular‐weight HA at a concentration of 24 mg/mL crosslinked with 1,4‐butanediol diglycidyl ether (BDDE) and have been on the market since 2000 (Table 1). They are marketed under different brand names in different jurisdictions with a range of indications. HYC‐24L (Juvéderm Ultra 2 and the equivalent Juvéderm Ultra XC) are the least cohesive formulations and can be used for correcting fine lines and wrinkles and for lip definition; HYC‐24L+ (Juvéderm Ultra 3, Juvéderm Ultra Smile, and the equivalent Juvéderm Ultra Plus XC) have intermediate properties and may therefore be appropriate for moderate lines and wrinkles and for lip augmentation; and Juvéderm Ultra 4 is the most cohesive formulation, designed for deep wrinkles and folds, as well as for cheekbone augmentation and lip enhancement.

**TABLE 1:** Key products and studies with the Juvéderm range.

Product	HA concentration (mg/mL)	*G*′_5Hz_ (Pa)	Cohesivity/Fn (gmf)	Unconstrained water uptake^a^ (%)	Intended use as per US DFU^b^	Target injection plane^b^	Manufacturer‐sponsored studies
Hylacross^c^							
HYC‐24 L	24	207	96	622	Facial wrinkles and folds (such as NLFs) Lips and perioral area	Mid to deep dermis/lip mucosa	NLFs^d^ [8, 9] Lips/perioral^d^ [10, 11]
HYC‐24 L+	24	263	112	454	Facial wrinkles and folds (such as NLFs)	Mid to deep dermis	Lips^e^ [12] NLFs^d^ [8, 9, 13, 14]
Vycross							
VYC‐12 L	12	166	12	< 100	Improvement of skin smoothness of the cheeks	Intradermal	Skin smoothness/quality [15, 16, 17, 18]
VYC‐15 L	15	271	19	133	Lip augmentation Correction of perioral rhytids Improvement of infraorbital hollowing	Intradermal (perioral, lips) or supraperiosteal (infraorbital hollowing)	Lips/perioral [19, 20, 21, 22, 23, 24] Infraorbital [25, 26]
VYC‐17.5 L	17.5	340	30	184	Facial wrinkles and folds (such as NLFs)	Mid to deep dermis	NLFs [27, 28, 29, 30] RCLs [31] Lips [32] Marionette lines [33] Forehead [34]
VYC‐20 L	20	398	40	227	Augmentation of cheeks, chin, and temples	Supraperiosteal or subcutaneous	Midface [35, 36, 37, 38, 39, 40, 41] Nose [42, 43] Chin [44] Temples [45]
VYC‐25 L	25	665	93	253	Improvement of jawline definition	Supraperiosteal or subcutaneous	Chin/jawline [46, 47, 48, 49, 50, 51]

^a^ These in vitro assessments represent the maximum (unconstrained) ability to absorb water, but once HA injectables are administered to patients, other factors will constrain their ability to expand (e.g., the water content and composition of surrounding tissues and the forces acting upon them) [5].

^b^ As per the US DFU but indications may vary in other countries.

^c^ With regard to HYC‐24L, Juvéderm Ultra XC in the US is equivalent to Juvéderm Ultra 2 in the European Union, and with regard to HYC‐24L+, Juvéderm Ultra Plus XC in the US is equivalent to Juvéderm Ultra 3 and Juvéderm Ultra Smile in the European Union.

^d^ Some of these studies were performed using the equivalent formulation without lidocaine [8, 9, 11, 14].

^e^ Study was performed with the formulation used in the European Union. DFU, Directions for Use; HA, hyaluronic acid; NLF, nasolabial fold; RCL, radial cheek line. Rheologic and physicochemical characteristics are taken from de la Guardia et al. [5].

The Vycross technology contains high‐molecular‐weight HA (> 500 kDa based on a patented mix of various lengths), crosslinked with BDDE, and formulated to different concentrations. Each of the formulations has been designed to generate a different set of rheological and physicochemical properties, facilitating usage across a range of indications—which may vary between countries (Table 1) [5]. For example, the Vycross product with the lowest HA concentration (VYC‐12L) has been shown to improve superficial cutaneous depressions (such as fine lines) and skin quality attributes like hydration and elasticity [15, 16, 52]. VYC‐20L has higher cohesivity and elastic modulus (*G*′), and is particularly suitable for volumizing and contouring of facial areas like the midface, nose, chin, and temples [35, 36, 37, 38, 39, 40, 41, 42, 43, 44, 45]. The Vycross gel with the highest HA concentration (VYC‐25L) is a robust, structural, injectable implant product with high levels of lifting capacity, associated with more defined and sharp contouring, as well as enhanced projection for areas like the chin and jawline, following subcutaneous or supraperiosteal injection [46, 47, 53, 54]. The other two products in the range—VYC‐15L and VYC‐17.5L—have intermediate properties to facilitate different levels of contouring and/or the treatment of skin depressions and wrinkles.

The first Vycross product came to market more than 15 years ago in Europe, followed a few years later by the US, and they are now available in ~100 countries worldwide. Hence, there is extensive experience with their clinical use. Data from pivotal trials have shown that these products are safe and effective in a multitude of indications, and yield natural outcomes as assessed by patients and clinicians [17, 19, 20, 21, 22, 27, 31, 32, 45, 48, 55, 56]. However, such studies were typically limited to 1–2 rounds of treatment and could not practically be continued over multiple cycles of repeat injections lasting 5–10 years or more.

There have been suggestions that long‐term use of HA injectables over many rounds of treatment can lead to progressive development of unnatural results, including surface irregularities, general disproportion or distortion of the face, and/or an overfilled appearance sometimes referred to as “pillow face” or “bread face” [56, 57]. This characterization is primarily a social‐media phenomenon, although it has also been discussed in the peer‐reviewed medical literature [56, 57, 58, 59]. Importantly, it could act as a deterrent to some patients and practitioners. However, in the authors' experience of following key best‐practice principles, long‐term HA injectable treatment can help to preserve patients' natural beauty and contribute to positive aging with natural outcomes.

Over the past 20 years, treatments with HA injectables have become highly skilled procedures that incorporate many different indications for refining, balancing, and better defining the face and neck. The aim of this paper is to provide technical expert considerations for long‐term use of Juvéderm products, and to showcase patient case studies treated for ≥ 10 years, demonstrating safe, precise, effective, and natural outcomes.

## 2 Methods

In July 2025, the six authors who are current aesthetic practitioners independently completed a written questionnaire on their clinical experiences of long‐term treatment with Juvéderm HA injectables. The questionnaire covered their level of usage; benefits of long‐term planning and treatment; considerations on achieving natural and safe outcomes across multiple rounds of injection; and patient management. The present paper collates the joint experience of these experts, representing diverse specialties (dermatology, ophthalmology, and aesthetic medicine) across six countries on five different continents around the world.

At the time of completing the questionnaire, they had all been using Juvéderm products in their clinical practice for between 17 and 26 years, apart from Y.S., who had used them for 9 years (reflecting the more recent approvals in China). Collectively, the authors have treated more than 30 000 patients with Juvéderm, of whom around 10 000 have been injected with these products for ≥ 10 years.

Case presentations are also provided for several patients who have received long‐term treatment with Juvéderm for ≥ 10 years (or ≥ 5 years for the patient from China due to the later approval dates). All of the patients whose photographs are used provided written informed consent.

To determine whether there were any previously published data on the outcomes of long‐term treatment with HA injectables, a literature search was performed in the PubMed database, up to and including September 1, 2025. The following search terms were used, with no limits applied: “hyaluronic acid” AND filler AND (“long term” OR repeat). This yielded a total of 268 unique hits. Review of these records identified no studies that assessed outcomes over ≥ 10 years of treatment with any HA product. There was one retrospective case review in which complications were assessed in patients followed up over ≥ 5 years of periorbital treatment with an HA gel [60]. The study found no severe complications across more than 1000 injections in 147 patients. However, treatment was not based on Juvéderm products and the study did not evaluate effectiveness outcomes (including naturalness), so it will not be considered further in the present paper.

### 2.1 Benefits of Long‐Term Treatment

Like many other injectable aesthetic treatments, HA gels are not designed to be permanent, as they are naturally biodegradable and therefore metabolized through normal degradation pathways. This means that periodic repeat injections are necessary if outcomes are to be maintained—but this can often have important advantages for patients and practitioners. For example, regular consultations allow for the development of strong and trusting patient–practitioner relationships that in some cases may last for decades. Frequent consultation also facilitates effective monitoring of safety, effectiveness, and patient satisfaction with results.

From a practical perspective, long‐term planning and treatment allow the healthcare professional to develop a full face and neck approach that progressively addresses key underlying anatomical concerns, as well as the complex and continuous changes that naturally occur with aging. Thus, the approach can be adapted over time as the patient's individual needs evolve.

In addition, long‐term treatment with HA injectables (alongside other relevant modalities if indicated) allows practitioners to fully leverage each product's physicochemical characteristics in a manner that has advantageous effects outside the explicit treatment zone. Such “indirect” benefits have been noted in several recent studies [61, 62, 63], and warrant further investigation in prospective studies.

Each of the six practitioners in the present author group was asked to list their top three reasons for choosing Juvéderm products when planning long‐term treatment with HA injectables (Table 2). Key themes related to the breadth of the product portfolio (allowing for different treatment effects across multiple anatomical layers), its proven safety profile over many years of usage, the predictability of outcomes, and the long‐lasting and natural character of results.

**TABLE 2:** Top three reasons for choosing Juvéderm HA injectables when planning long‐term treatment.

Author^a^	Top three reasons for choosing Juvéderm		
R.A.	Predictable outcomes	Long‐lasting natural results	Safety profile
G.C.	Safety profile	Precision	Support from manufacturer
S.R.	Predictable outcomes	Long‐lasting natural results	Ability to address multiple anatomical layers
Y.S.	Structural restoration and lifting effects	Long‐lasting natural results	Safety profile
F.U.G.	Predictable outcomes	Broad portfolio^b^	Safety profile
S.G.Y.	Effectiveness	Long‐lasting natural results	Broad portfolio^b^

^a^ Listed in alphabetical order by surname.

^b^ Product range with widely varying rheologies and HA concentrations. HA, hyaluronic acid.

### 2.2 Ensuring Natural and Safe Outcomes During Long‐Term Treatment: Key Principles of Best Practice

Data from a number of studies have shown that Juvéderm products are associated with natural outcomes across 1–2 treatment cycles when best‐practice methods are employed [10, 17, 19, 20, 21, 22, 27, 31, 32, 45, 48, 55]. For example, in a recent randomized controlled trial of VYC‐20L for the improvement of temple hollowing, ~90% of patients were *satisfied* or *very satisfied* with how natural the temple area looked and felt at 3 months post‐treatment [45]. Similarly, positive patient‐reported naturalness outcomes have been demonstrated in recent studies of VYC‐15L for infraorbital hollowing [55] and VYC‐17.5L for lip augmentation [32].

In a social perception study, perceived naturalness before and after combined pan‐facial treatment with Juvéderm HA fillers and onabotulinumtoxinA was assessed by a group of nonclinical blinded observers [64]. Importantly, these observers were unaware that participants had received aesthetic treatment, and the “before” and “after” footage of the same participant were never viewed by the same observer. Ratings of naturalness were found to be significantly improved after treatment as compared with before (*p* = 0.016).

**FIGURE 1:**
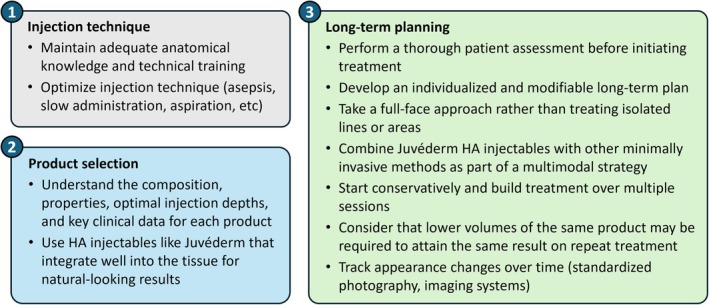
Key factors in achieving natural long‐term results with Juvéderm HA injectables. HA, hyaluronic acid.

Maintaining natural outcomes across multiple cycles of HA injectable treatment over many years requires clinicians to optimize their practice and techniques. In the authors' experience, key factors can be summarized according to: (i) injection technique; (ii) product selection; and (iii) long‐term planning (Figure 1).

With regard to the fundamentals of good technique with Juvéderm products, these have been widely discussed elsewhere, for example in recent expert considerations on VYC‐25L [53] and VYC‐12L [65]. These principles are essential to gaining natural results and, even more importantly, for optimizing patient safety. They include consideration of the optimal injection device and appropriate target tissue layer, slow product administration, deposition of small amounts in each injection area, strict adherence to principles of aseptic technique, the use of aspiration as a helpful safety checkpoint, and careful monitoring for early signs of vascular compromise and other adverse events [53, 65, 66, 67]. Notably, all of the Juvéderm products come with an improved ergonomic syringe (relative to its predecessor) that facilitates the injection procedure (e.g., easier aspiration, improved gauging of administration volume, and greater injector comfort [68]), and could contribute to optimizing precision and safety.

Natural results are more likely to be achieved if clinicians avoid common errors like administration into the wrong plane for the desired effect, the use of overly large bolus injections, or excessive overall volumes [56, 57, 58, 59]. Patients may sometime request more product, but practitioners should use their clinical judgment to decide whether or not this is appropriate and refuse if necessary. In addition, it is essential that practitioners maintain an in‐depth knowledge of facial anatomy. Inadequate understanding of key anatomical differences between patient subtypes (e.g., based on ethnicity) has been suggested as a key cause of overfilling and hence of unnatural results [57]. Treatment should always be tailored to the individual patient, based on enhancing appearance without detrimentally impacting personal identity. Furthermore, clinicians should always seek adequate technical training before treating their own patients.

With respect to appropriate product selection within the Juvéderm range, this first requires that practitioners appreciate the individual compositions, physicochemical/rheological properties, optimal injection depths, and key clinical data relating to each product (Table 1). These characteristics are central to HA gel performance in situ [5], and should always be considered alongside the anatomy and treatment goals of the individual patient. Incorrect product selection will most likely increase the risk of unnatural outcomes. For example, a structural product like VYC‐25L should be injected deep into the subcutaneous or supraperiosteal plane, and overly superficial placement risks both visibility and irregularity [53].

In broader terms, it is also important to use HA products that integrate well into the tissue as this may help to promote natural results. Notably, even the most robust and structural of the Juvéderm gels—VYC‐25L—has been shown by ultrasound examination to integrate totally into the tissue within 30 days of injection [69].

Long‐term treatment planning is particularly crucial to avoiding the gradual development of unnatural results over repeat treatment, and practitioners are advised to maintain a 360° approach to patient care [70]. This should always begin with a thorough assessment, based on developing a complete understanding of the patient's aesthetic concerns, medical history, and treatment goals; implementation of a comprehensive, multi‐tissue assessment of the full face and neck; consideration of all available treatments and techniques; and development of a long‐term, modifiable, individualized treatment timeline [70]. This approach reflects the key importance of considering the whole face and neck—and not just treating isolated lines or areas—to create harmonious results. Clinicians should avoid chasing specific lines and instead focus on restoring facial proportions and achieving an overall balanced look.

The 360° model also reflects the value of combining HA injectables as part of a multimodal strategy, alongside other minimally invasive methods, such as neuromodulators, biostimulators, and energy‐based devices. Multimodal treatment allows the targeting of multiple tissue layers and facilitates potential mechanistic synergies [65, 71]. Such an approach might also help to minimize the volume of HA gel required, whereas using excessive amounts alone may sometimes contribute to unnatural outcomes [57].

A further aspect of treatment planning that could help to foster natural results with HA injectables in the medium‐to‐long term is to start with a conservative approach [56]. The patient can then be re‐assessed at subsequent visits, with additional HA injected if appropriate to achieve optimal outcomes. Administration of modest amounts of product at regular intervals, based on carefully agreed goals, gives the best chance of avoiding overfilling.

In addition, clinicians should consider the possibility that lower volumes of the same product may be required to attain the same result on repeat treatment. A possible reason for this is that, although HA injectables degrade naturally over time, imaging studies suggest there may sometimes be a residual presence in the tissue for 2 years or more [72, 73, 74, 75, 76]. Moderating quantities over long‐term treatment can also have the indirect benefit to patients of better distributing overall costs.

A final key consideration is the tracking of results over time. In particular, it is important to take high‐quality standardized photographs every time the patient is treated (e.g., consistent angles and lighting, plain background, free from make‐up [67]). This allows changes in facial proportions to be accurately monitored, and the risk of a gradual drift in patient or clinician perception of “natural” can therefore be minimized [77]. This tracking process can be further enhanced using quantitative volumetric analyses with 2D and 3D imaging systems.

By following these principles, long‐term natural results can be achieved and overfilling avoided. In the unlikely event that patients do experience unnatural outcomes, appropriate management protocols should be invoked. In particular, HA can be effectively hydrolyzed with hyaluronidase. For example, Schelke and colleagues recently described the use of facial ultrasound to precisely locate implanted HA and guide the administration of hyaluronidase in 28 cases of overfilling; patients and physicians noted immediate improvements in all instances [78]. However, large‐scale dissolution of HA gels with hyaluronidase can also have unwanted consequences—such as facial hollowing and reduced skin elasticity—possibly compounded by accelerated degradation of endogenous HA [79, 80]. Such procedures should therefore be performed only after careful consultation with the patient.

### 2.3 Managing Patient Concerns

New patients sometimes present with significant concerns about unnatural results with HA injectables and may be reluctant to receive treatment. If these products are indicated, it might therefore be necessary to assuage the patient's fears. There are a number of straightforward strategies that can help to achieve this.

First, it is important to build a strong connection with the patient. By listening to them carefully and understanding their wishes, the practitioner can then begin to describe the treatment requirements and discuss HA injectables if indicated—noting their ability to effect subtle changes for a refreshed and healthy appearance. Second, simple educational methods should be employed based on explaining the aging process and discussing how HA injectables work to counteract this. For example, it may be worth noting that HA is a naturally occurring substance in the skin that has a role in collagen stimulation [81, 82], and because levels tend to decline with age, there is value in replenishment. In addition, it is important to show before‐and‐after images that demonstrate long‐term natural results in other patients. With some individuals, it may also be worthwhile to share clinical data confirming the naturalness of outcomes with Juvéderm products. Third, explaining to the patient that implanted HA can be degraded in situ using hyaluronidase provides reassurance that the procedure is reversible if they are unhappy with results. Fourth, if the patient is then willing to try HA injectable treatment, they should be involved in the planning process. This further develops trust and confidence and helps to ensure that their expectations are realistic. The injection plan should always be fully explained, including the timings and rationale for each step. Finally, as already described with regard to treatment itself, it may be advisable to start gradually and build from there, spreading treatment across multiple sessions if needed.

### 2.4 Long‐Term Juvéderm Treatment: Clinical Cases

**FIGURE 2:**
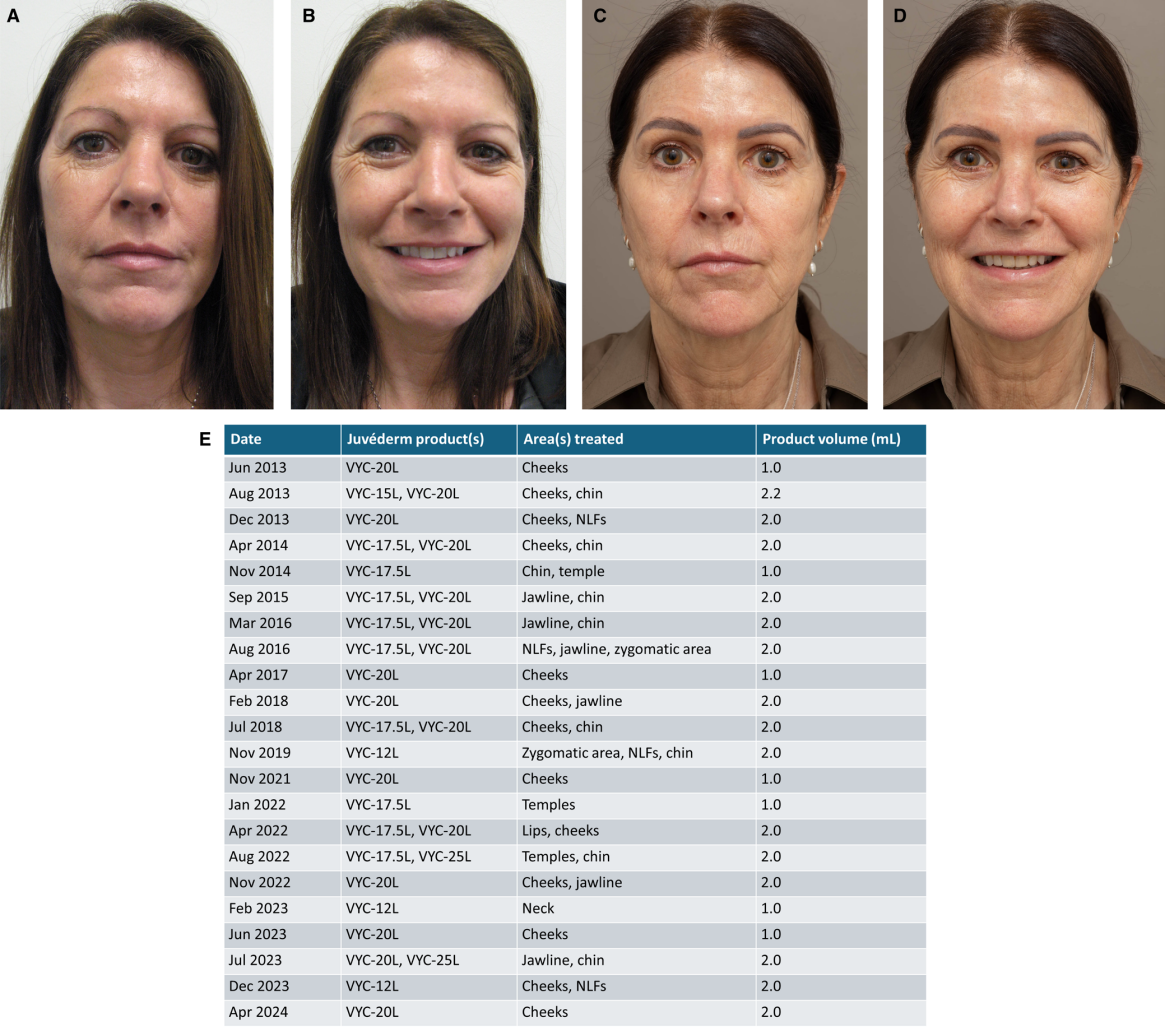
Long‐term natural results with Juvéderm used for 12 years. Parts A and B show a female patient aged 48 years, prior to treatment with Juvéderm products. Parts C and D show her 12 years later—aged 60 years—following multiple rounds of injections (1 year since the most recent). Her treatment plan is summarized in part E. Over this period, she also received long‐term neuromodulator injections (UFLs, DAO, chin), as well as occasional treatment for submental fat reduction using DCA, and skin quality treatment of the face and neck using a non‐Juvéderm HA product. Patient images are courtesy of author S.R. DAO, depressor anguli oris; DCA, deoxycholic acid; HA, hyaluronic acid; NLF, nasolabial fold; UFL, upper facial line.

**FIGURE 3:**
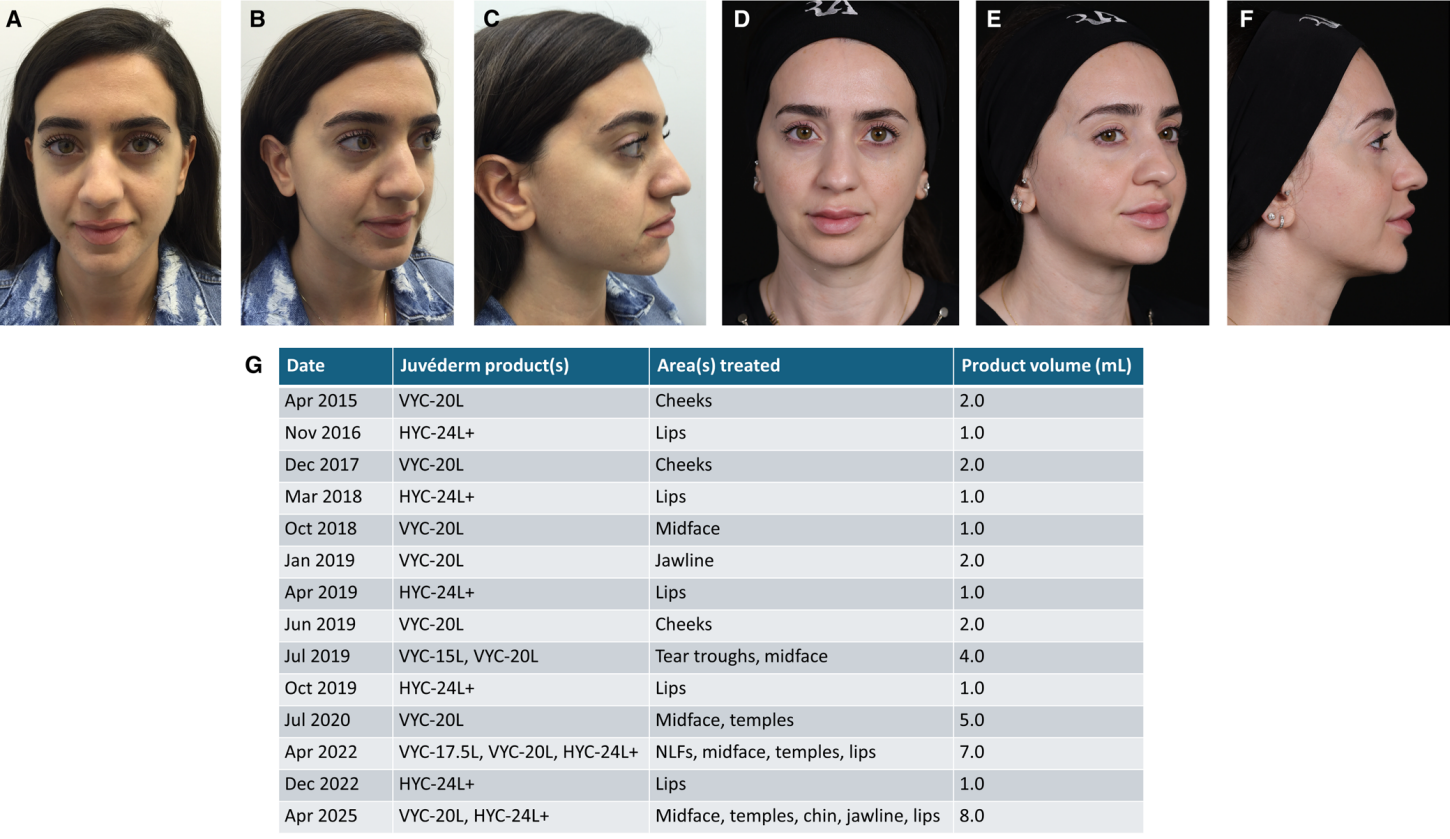
Long‐term natural results with Juvéderm used for 10 years. Parts A–C show a female patient aged 25 years, prior to treatment with Juvéderm products. Parts D–F show her 10 years later—aged 35 years—following multiple rounds of injections (2 months since the most recent). Her treatment plan is summarized in part G. Over this period, she also received long‐term neuromodulator injections for upper facial lines. Patient images are courtesy of author R.A. NLF, nasolabial fold.

**FIGURE 4:**
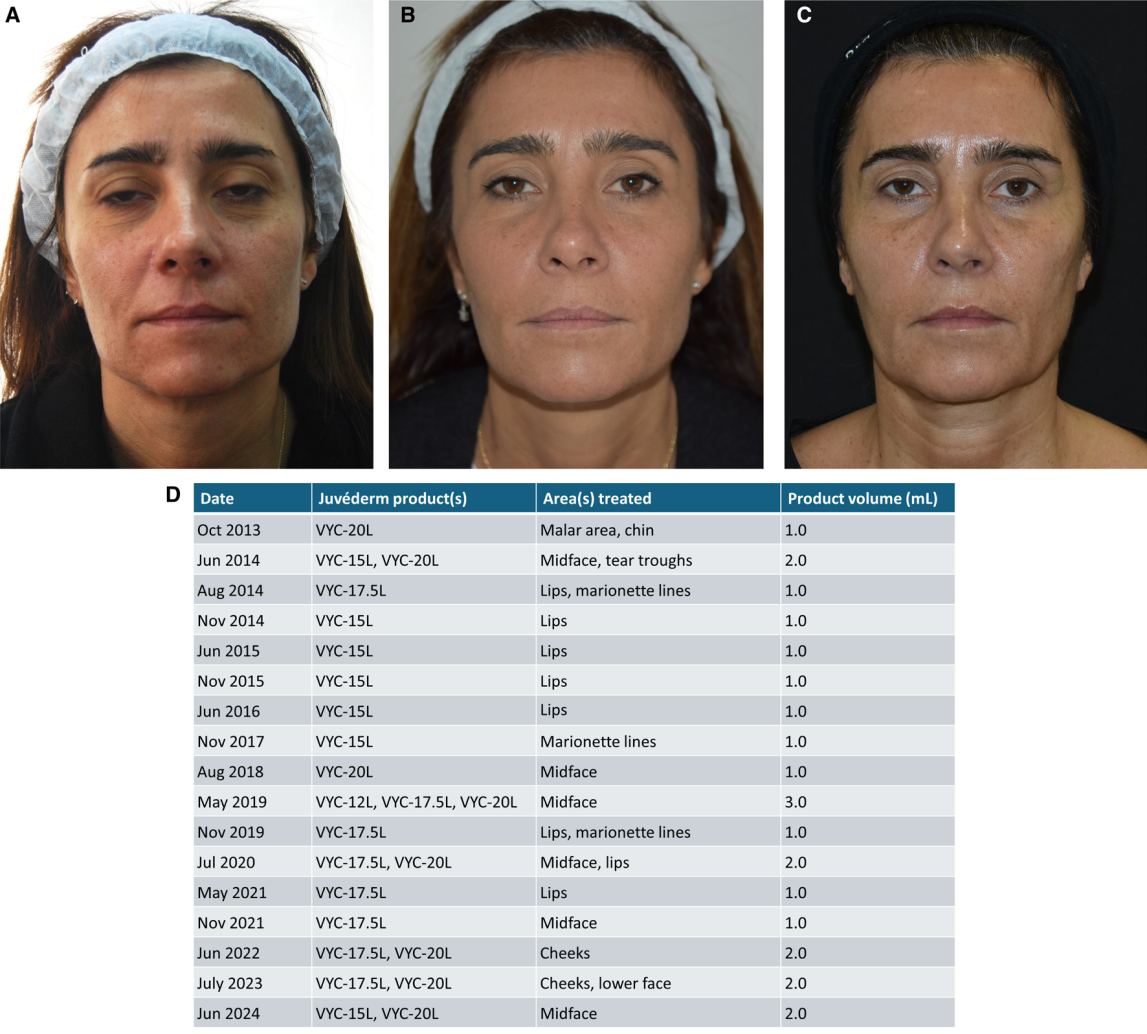
Long‐term natural results with Juvéderm used for 11 years. Part A shows a female patient aged 44 years, prior to treatment with Juvéderm products. Part B shows her 4 years later, and part C shows her 12 years later—aged 56 years—following multiple rounds of injections (1 year since the most recent). Her treatment plan is summarized in part D. Over this period, she also received long‐term neuromodulator injections (UFLs, DAO, platysma, chin) and intense‐pulsed light treatment, as well as occasional microfocused ultrasound. Patient images are courtesy of author G.C. DAO, depressor anguli oris; UFL, upper facial line.

**FIGURE 5:**
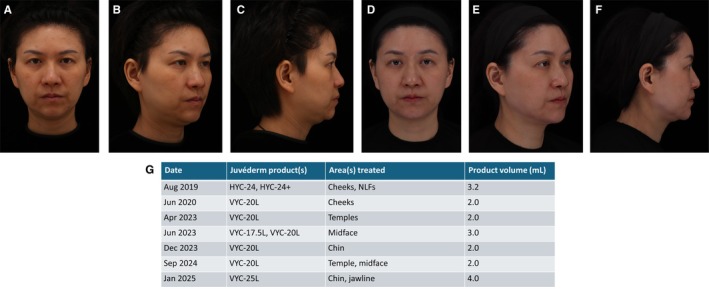
Long‐term natural results with Juvéderm used for 5.5 years. Parts A–C show a female patient aged 40 years, prior to treatment with Juvéderm products. Parts D–F show her 5.5 years later—aged 46 years—following multiple rounds of injections (photographs taken immediately after her most recent injection session). Her treatment plan is summarized in part G. Positive initial outcomes with Hylacross products (HYC‐24 and HYC‐24+) rapidly established patient confidence, and hence when the Vycross range was subsequently launched in China, she was happy to adopt the new formulations. Over this period, she also received many rounds of neuromodulator injections for upper facial lines; energy‐based device treatments for melasma, skin tightening, and skin quality improvement; and mesotherapy for skin quality. Patient images are courtesy of author Y.S. NLF, nasolabial fold.

Figures 2, 3, 4, 5 show patients treated over many years with Juvéderm injectables (mostly for ≥ 10 years). During this period, clinical practice in aesthetic medicine has undergone a fundamental transformation, moving away from a narrow focus on individual lines towards comprehensive enhancement of the full face and neck. The pre‐treatment images were mostly taken prior to the contemporary age of strict photographic standardization, but these cases nonetheless demonstrate natural long‐term results.

Figure 2 shows a patient treated for 12 years with various HA injectables from the Vycross range. Treatment was focused primarily on the cheeks, jawline, and chin. Overall, the patient looks refreshed, healthy, and natural. Furthermore, continued treatment over many years—based on modest product volumes of around 3 mL per year—has had a significant impact on her aging process, leading to an improved aesthetic outcome. Her face remains particularly harmonious in terms of upper, middle, and lower third proportions. The patient has also benefited from co‐treatment with other minimally invasive modalities, including neuromodulator and HA‐based skin quality treatments, and her skin texture is notably improved.

Figure 3 shows an individual treated for 10 years with both Hylacross and Vycross injectables. She was initially injected using only small amounts of product, but greater quantities have been administered in more recent sessions—focusing particularly on the midface, temples, jawline, and lips. She looks healthy and natural, and shows improved midface structure that gives her a more relaxed appearance. In addition, the patient looks less tired because of improvements in the midface and tear trough areas. She also has better facial proportions, fuller temples, lips that are fuller and more contoured, and smoother nasolabial folds.

Figure 4 shows a patient treated for 11 years with HA injectables from the Vycross range. Her aim throughout was to improve facial harmony while maintaining her overall image and individuality. Thus, modest quantities were used throughout (~2 mL per year on average), primarily targeted at the lips and cheeks/midface. The photographs demonstrate that after more than a decade of treatment, the patient has attained balanced overall proportions, preserved facial structure, and an enhancement of her natural beauty.

In China, products from the Juvéderm range have only been approved in the past few years, and hence there are not many patients who have been treated for 10 years or more. However, Figure 5 shows an individual who was injected regularly with Hylacross and Vycross products for 5.5 years, as part of a multimodal plan incorporating neuromodulator, energy‐based device treatment, and mesotherapy. Injections were focused particularly on restoring structure to her midface, chin, and jawline. Prior to that, the patient had undergone double eyelid surgery and had received treatment with other HA injectables for nasolabial fold correction, but this did not help her achieve her goal of a more youthful and attractive appearance. By contrast, midface enhancement with VYC‐17.5L and VYC‐20L has yielded sustained improvements. Furthermore, her congenital mandibular hypoplasia has been corrected, most recently using VYC‐25L. HA injectables are among a small number of nonsurgical options for achieving such effects, and in this case resulted in more balanced facial proportions. She was in her 40s throughout—a time when women are undergoing significant hormonal changes—but regular treatment has nonetheless made her appearance seem softer, more feminine, and more attractive. The lifting capabilities of the products used are clearly demonstrated, but the results still appear very natural.

Overall, the four cases demonstrate some of the key principles discussed in this paper for ensuring natural long‐term results, including the value of a conservative initial approach followed by regular “maintenance” with small product volumes. These cases also highlight the importance of a full‐face strategy based on several treatment modalities as opposed to HA injectables alone.

### 2.5 Limitations and Future Work

This paper synthesizes the extensive knowledge and experience of long‐term treatment with Juvéderm injectables for ≥ 10 years from an international group of physicians. Nonetheless, a key limitation is the current lack of clinical data gathered over multiple rounds of treatment with these products (or with any other range of HA injectables). There remains a need to collect and publish such data from routine daily practice. Ideally, such studies should include specific evaluations of “naturalness” as assessed by both practitioners and patients. For example, the FACE‐Q Aesthetics module includes three different validated tools allowing patients to self‐report on the degree of importance they place pre‐injection on natural results and their perceptions of naturalness following aesthetic treatment [83, 84].

## 3 Conclusions

In the authors' experience, Juvéderm HA injectables can provide excellent, natural outcomes over long‐term use lasting ≥ 10 years, and they are well tolerated. However, it is important that practitioners follow key principles of best practice, including adherence with technical fundamentals, optimal product selection, and long‐term planning. Clinicians are most likely to maintain the naturalness of results over multiple rounds of injection if they consider the whole face and not just individual areas. It is advisable to start conservatively and build up from there, combine HA products with other treatments as part of a multimodal approach when indicated, and track changes in the patient's appearance over time.

## Author Contributions

All authors contributed to the conception and design of the paper and the interpretations provided; were involved in drafting the manuscript and revising it critically for important intellectual content; gave final approval of the version to be published; and agreed to be accountable for all aspects of the work.

## Funding

Writing and editorial assistance was provided to the authors by Dr. Timothy Ryder from Biological Communications Limited (London, United Kingdom) and funded by Allergan Aesthetics, an AbbVie company. Neither honoraria nor payments were made for authorship.

## Ethics Statement

The authors have nothing to report.

## Consent

Written informed consent were provided by the patients whose photographs are used in this publication.

## Conflicts of Interest

Yuexing Song is a speaker, trainer, and consultant for Allergan Aesthetics, an AbbVie company. Rami Abadi is a speaker and trainer for Allergan Aesthetics, an AbbVie company. Gabriela Cella is a speaker for Allergan Aesthetics, an AbbVie company, and for Galderma. Stefania Roberts is a speaker for Allergan Aesthetics, an AbbVie company, and for Dermocosmetica. Fernando Urdiales‐Gálvez is a speaker, trainer, and consultant for Allergan Aesthetics, an AbbVie company. Steve Yoelin is a speaker and consultant, and has conducted paid research for Allergan Aesthetics, an AbbVie company. Carola de la Guardia is an employee of Allergan Aesthetics, an AbbVie company.

## Data Availability

AbbVie is committed to responsible data sharing regarding the clinical trials we sponsor. This includes access to anonymized, individual, and trial‐level data (analysis data sets), as well as other information (e.g., protocols, clinical study reports, synopses, or statistical analysis plans), as long as the trials are not part of an ongoing or planned regulatory submission. These clinical trial data can be requested by any qualified researchers who engage in rigorous, independent, scientific research and will be provided following review and approval of a research proposal, Statistical Analysis Plan (SAP), and execution of a Data Use Agreement (DUA). Data requests can be submitted at any time after approval in the US and Europe and after acceptance of this manuscript for publication. The data will be accessible for 12 months, with possible extensions considered. For more information on the process or to submit a request, visit the following link: https://vivli.org/ourmember/abbvie/ then select “Home.”
